# Strategiewechsel zur minimal-invasiven Ösophagektomie – Ergebnisse an einem zertifizierten Zentrum

**DOI:** 10.1007/s00104-021-01550-2

**Published:** 2021-12-21

**Authors:** Felix Merboth, Jasmin Hasanovic, Daniel Stange, Marius Distler, Sandra Kaden, Jürgen Weitz, Thilo Welsch

**Affiliations:** 1 Klinik und Poliklinik für Viszeral‑, Thorax- und Gefäßchirurgie, Universitätsklinikum Carl Gustav Carus, Fetscherstr. 74, 01307 Dresden, Deutschland; 2 Klinik-Apotheke, Universitätsklinikum Carl Gustav Carus, Dresden, Deutschland

**Keywords:** Roboter-assistierte minimal-invasive Ösophagektomie, Darmdekontamination, Propensity Score Matching (PSM), Komplikationsrate, Krankenhausverweildauer, Robot-assisted minimally invasive esophagectomy, Bowel decontamination, Propensity Score Matching (PSM), Complication rate, Length of stay

## Abstract

**Hintergrund:**

Es gibt Hinweise, dass die roboterassistierte minimal-invasive Ösophagektomie (RAMIE) die Morbidität im Vergleich zur konventionellen Operationstechnik verringern kann.

**Ziel der Arbeit:**

Es erfolgte eine Vergleichsanalyse eines Single-Center-Strategiewechsels des Standards von offener Ösophagektomie zu RAMIE mit perioperativer, enteraler, selektiver Darmdekontamination (SDD).

**Material und Methoden:**

Patienten- und Morbiditätsdaten nach elektiver RAMIE entsprechend dem neuen Standardmanagement zwischen Juli 2018 und September 2020 wurden retrospektiv mit einer historischen Kontrollkohorte nach offener Ösophagektomie zwischen Januar 2014 und Juni 2018 verglichen. Es erfolgte eine 1:1-Propensity-Score-Matching(PSM)-Analyse.

**Ergebnisse:**

Insgesamt 75 Patienten konnten nach PSM in beiden Gruppen analysiert werden. Etwa zwei Drittel der Operationen erfolgte aufgrund eines Adenokarzinoms und ein Drittel bei Plattenepithelkarzinom. Im Median wurden 22 bzw. 21 Lymphknoten reseziert. Die intrathorakale Ösophagogastrostomie erfolgte in der RAMIE-Gruppe in 97 % mit einem Zirkularstapler mit ≥28 mm Durchmesser (offen: 25 mm in 90 % der Fälle). Die Operationszeit war länger (Median 490 vs. 339 min, *p* < 0,001), hingegen waren der Blutverlust (Median 300 vs. 500 ml, *p* < 0,001), die Anastomoseninsuffizienz- (8,0 % vs. 25,3 %, *p* = 0,004), Wundinfektions- (4,0 % vs. 17,3 %, *p* = 0,008) und pulmonale Komplikationsrate (29,3 % vs. 44,0 %, *p* = 0,045) sowie die mediane Krankenhausverweildauer (14 vs. 20 Tage, *p* < 0,001) und die 90-Tage-Mortalität signifikant geringer verglichen mit der offenen Kontrollkohorte (4,0 % vs. 13,3 %, *p* = 0,039).

**Diskussion:**

Ein konsequenter Wechsel des perioperativen Managements u. a. mit RAMIE und SDD kann zu einer stabilen Reduktion der Morbidität ohne Einschränkungen der onkologischen Radikalität führen.

## Abstract (en)

**Background:**

There are indications that robot-assisted minimally invasive esophagectomy (RAMIE) can reduce the morbidity compared with the conventional operative technique.

**Objective:**

A comparative analysis of a single-center change in strategy of the standard from open esophagectomy to RAMIE with perioperative, enteral, selective bowel decontamination (SBD) was carried out.

**Material and methods:**

Patient and morbidity data after elective RAMIE treated according to the novel standard management between July 2018 and September 2020 were compared retrospectively with an historical control cohort after open esophagectomy between January 2014 and June 2018. A 1:1 propensity score matching (PSM) analysis was performed.

**Results:**

A total of 75 patients could be analyzed in both groups after PSM. Approximately two thirds of the operations were carried out due to an adenocarcinoma and one third due to a squamous cell carcinoma. The median number of resected lymph nodes was 22 and 21, respectively. In the RAMIE group the intrathoracic esophagogastrostomy was performed using a circular stapler with a diameter of ≥28 mm in 97%, whereas a 25 mm stapler was used in 90% in the control group. The operative time was longer (median 490min vs. 339 min, *p* < 0.001) but in contrast blood loss (median 300ml vs. 500 ml, *p* < 0.001), anastomotic leaks (8.0% vs. 25.3%, *p* = 0.004), surgical site infections (4.0% vs. 17.3%, *p* = 0.008) and pulmonary complication rates (29.3% vs. 44.0%, *p* = 0.045) as well as the median hospital stay (14 days vs. 20 days, *p* < 0.001) and 90-day mortality were significantly reduced compared with the open control group (4.0% vs. 13.3%, *p* = 0.039).

**Conclusion:**

A consistent change of the perioperative management including RAMIE and SBD can lead to a stable reduction of morbidity without compromising oncological radicalness.

Das Ösophaguskarzinom gehörte 2020 zu den zehn häufigsten Krebserkrankungen und zeigt in den letzten Jahren eine zunehmende Inzidenz vor allem in westlichen Ländern [23]. Bei resektablen Befunden wird heute in der Regel ein multimodaler Therapieansatz mit (neo‑)adjuvanter (Radio‑)Chemotherapie und Operation verfolgt. Die Resektion ist bei kurativer Therapieintention ein elementarer Outcomeprädiktor [4]. Die Ösophagusresektion zählt jedoch zu den komplexesten viszeralchirurgischen Eingriffen und ist trotz moderner Operationstechniken mit einer hohen Morbidität und Mortalität verbunden [1]. Aktuelle randomisierte Studien belegen eine potenzielle Überlegenheit der minimal-invasiven Operationsmethode mit geringerer Komplikationsrate [2, 14, 22].

## Hintergrund

Die Technikentwicklung der Ösophagektomie wird wesentlich durch die onkologische Radikalität und Morbidität bestimmt. Anastomoseninsuffizienzen (AI) der Ösophagogastrostomie sowie Pneumonien sind häufige und schwerwiegende postoperative Komplikationen nach Ösophagusresektion. In den vergangen Jahren konnte gezeigt werden, dass besonders diese Komplikationen durch die Einführung minimal-invasiver Ösophagektomieverfahren (MIE) im Vergleich zu offenen Operationsverfahren deutlich reduziert werden konnten [2, 14, 22].

Entscheidend für das Outcome scheint auch die Anastomosentechnik zu sein. So erwies sich in mehreren Studien die Rekonstruktion nach Ivor Lewis mit Magenschlauchhochzug und hoher intrathorakaler Zirkularstapleranastomose im Vergleich zu anderen Rekonstruktionstechniken als überlegen [5, 26]. Aufgrund der dreidimensionalen Sicht, einer zitterfreien Bewegungsübertragung und dem größeren Freiheitsgrad der Instrumente im Thorax und Mediastinum sind roboterassistierte Verfahren den konventionellen minimal-invasiven Verfahren möglicherweise überlegen [11]. Die roboter-assistierte minimal-invasive Ösophagektomie (RAMIE) ergab zudem bei relativ niedriger Morbidität und Mortalität exzellente onkologische Ergebnisse [20].

Eine weitere Option zur Reduktion infektiöser Komplikationen nach Ösophagusresektion ist die perioperative orale Prophylaxe mit topischen Antiinfektiva, wie sie bereits in der kolorektalen Chirurgie weitgehend akzeptiert ist [18]. Diese sog. selektive Darmdekontamination (SDD) konnte in einer aktuellen Metaanalyse das Auftreten von Anastomoseninsuffizienzen und Pneumonien nach Operationen am oberen Gastrointestinaltrakt signifikant reduzieren [19].

Im Juli 2018 erfolgte in unserer Klinik aufgrund der überzeugenden Datenlage in der Literatur eine konsequente Umstellung des perioperativen Managements bei elektiven Ösophagusresektionen:

- primärer Operationsansatz mittels roboter-assistierter minimal-invasiver Ösophagektomie (RAMIE) mit intrathorakaler End-zu-Seit-Ösophagogastrostomie (Ivor Lewis) als Zirkularstapleranastomose,
- Verwendung einer Zirkularstaplergröße von mindestens 28 mm Durchmesser,
- Überprüfung der Magenschlauchperfusion mittels Indocyaningrünfluoreszenz (ICG) im Abdominalteil mit Markierung der Perfusionsgrenze und erneut nach intrathorakaler Anastomosenanlage,
- Übernähung der Stapleranastomose zirkulär fortlaufend mit resorbierbarem Nahtmaterial (4-0),
- perioperative, enterale, selektive antiinfektive Gastrointestinaltraktdekontamination (SDD) zusätzlich zur perioperativen i.v. Standardantibiotikaprophylaxe

Im Zeitraum zuvor wurden Patienten primär konventionell abdominal und thorakal operiert, mit der Ausnahme selektionierter Patienten, die für einen minimal-invasiven Zugang geeignet schienen. In dieser Phase erfolgten auch an selektionierten Patienten RAMIE-Eingriffe mittels intrathorakaler Seit-zu-Seit-Ösophagogastrostomie. Alle Eingriffe in diesem Zeitraum erfolgten ohne SDD, in der Regel mit einer Staplergröße von 25 mm Durchmesser und Übernähung der Anastomose mit zirkulär angeordneten U‑Nähten.

## Methodik

### Studiendesign

Es handelt sich um eine retrospektive Studie mit einer historischen Vergleichskohorte. Berücksichtigt wurden für die Interventionsgruppe (RAMIE) alle konsekutiven Patienten, die sich im Zeitraum von Juli 2018 bis September 2020 an der Klinik und Poliklinik für Viszeral‑, Thorax- und Gefäßchirurgie des Universitätsklinikums Carl Gustav Carus zur elektiven Ösophagektomie vorstellten. Diese Patienten wurden entsprechend nach den o. g. neuen Standards behandelt. In dieser Studie wurden alle Patienten aus dem genannten Zeitraum analysiert, bei denen mindestens der thorakale Teil oder aber die vollständige Operation roboterassistiert erfolgte. Die Studie wurde von der zuständigen Ethikkommission an der Technischen Universität Dresden zustimmend bewertet (BO-EK-38012021).

Alle Patienten im Interventionszeitraum haben zusätzlich zur Teilnahme an der multizentrischen German-Xi-Registerstudie eingewilligt, deren Ergebnisse separat publiziert werden sollen (EK296072017). Im Rahmen dieser Registerstudie erfolgte eine prospektive Erfassung der Patienten- und Operationsdaten.

Als historische Vergleichskohorte wurden Patientendaten aus dem Beobachtungszeitraum Januar 2014 bis Juli 2018 (konventionelle Operationsgruppe, im Folgenden: „Kontrolle“) analysiert. Es erfolgte eine 1:1-Propensity-Score-Matching-Analyse.

### Perioperatives Management

Seit der Umstellung des perioperativen Managements bei elektiven Ösophagusresektionen im Juli 2018 ist das Standardverfahren eine RAMIE nach Ivor Lewis entsprechend den o. g. Standards, wobei sowohl im abdominellen als auch im thorakalen Teil der Operationsroboter eingesetzt wird [7]. Im Fall ausgeprägter Adhäsionen nach Voroperationen oder bei Tumorinfiltration in umgebende Strukturen erfolgte ein angepasstes Hybridverfahren (hRAMIE) mit konventionell offener Operation am Abdomen oder Thorax und robotischer Komplettierung des jeweils zweiten Teils.

Neben der Umstellung der operativen Technik erfolgte ebenfalls die Einführung einer prophylaktischen perioperativen SDD nach mechanischer Darmreinigung. Die SDD-Lösung besteht aus zwei Komponenten, die beide gemeinsam verabreicht wurden: eine Suspension mit Colistin, Tobramycin und Amphotericin B (angefertigt durch die Klinikapotheke des Universitätsklinikums Carl Gustav Carus; [16]) sowie eine Vancomycin-Lösung oder -Kapseln („off-label use“). Patienten erhielten am Vortag der Operation eine mechanische Darmreinigung mit 3 l Darmspüllösung und 2 Dosen SDD am Vorabend. Nach Anlage der Ösophagogastrostomie erfolgte eine erste lokale SDD-Gabe von 20 ml im Bereich der Anastomose über eine Magensonde. Postoperativ wurden täglich 4 Dosen oral bis zum 5. postoperativen Tag eingenommen.

### Operationstechnik

Die detaillierten Operationsschritte eines standardisierten RAMIE-Eingriffes mittels des 4‑armigen DaVinci-Xi™-Operationsroboters folgten einer veröffentlichten Technik [7, 8]. Die Operationen wurden insgesamt von 3 Operateuren mit Erfahrung in der roboterassistierten Chirurgie bei anderen viszeralchirurgischen Resektionen durchgeführt. Ein überlappendes Proctoring während der ersten ca. 20 RAMIE-Fälle erfolgte bei der Einführung je eines weiteren Operateurs.

Zusammengefasst befindet sich der Patient für den abdominellen Teil in Rückenlage und 15- bis 20°-Anti-Trendelenburgposition. Die 4 Robotertrokare werden in einer horizontalen Linie oberhalb des Bauchnabels eingebracht zusammen mit einem weiteren Assistententrokar und einem Leberretraktor. Sämtliche abdominellen Operationsschritte erfolgen robotisch. Grundsätzlich erfolgt bei maligner Grunderkrankung eine D2-Lymphadenektomie. Der Magenschlauch wird mit endoskopischen Linearstaplern mit einer Breite von 4,5 cm gestielt an der Arteria gastroepiploica und gastrica dextra gebildet. Eine Perfusionskontrolle wird mittels ICG durchgeführt und die Perfusionsgrenze mit Naht oralseitig markiert. Standardmäßig wird auf abdominelle Drainagen verzichtet.

**Figure 1:**
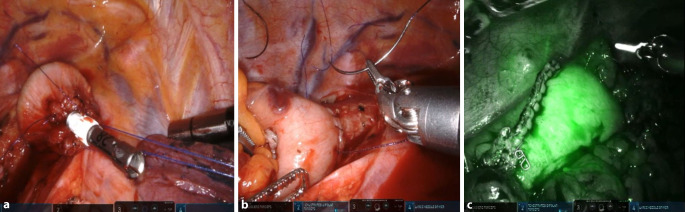

Für den thorakalen Teil werden die Patienten in eine überdrehte Linksseitenlage unter Einlungenventilation positioniert. Die 4 Robotertrokare werden auf einer geschwungenen Linie zwischen 4. und 10. Interkostalraum (ICR) eingebracht. Der Assistententrokar wird weiter ventral im 5. ICR etabliert. Die intrathorakale Resektion erfolgt en bloc mit der Lymphadenektomie. Der Ductus thoracicus wird selektiv dargestellt und geclippt. Nach Durchtrennung des Ösophagus oberhalb der V. azygos und Schnellschnittuntersuchung des Resektionsrandes folgt das Einbringen der Staplerandruckplatte und das Bergen des Resektats über eine Minithorakotomie im 5. ICR. Darüber wird anschließend die Ösophagogastrostomie angelegt und robotisch fortlaufend zirkulär übernäht (Abb. 1).

### Morbiditätsgrading

Die Morbiditätsanalyse wurde von 2 Ärzten unserer Klinik unabhängig voneinander anhand der Patientenakten durchgeführt. Dabei wurden postoperative Komplikationen mithilfe der Klassifikation nach Clavien und Dindo (CDC) erfasst [3, 6], welche durch die Japan Clinical Oncology Group konkretisiert wurden [10]. Diese Komplikationen wurden dokumentiert, wenn sie innerhalb von 90 Tagen postoperativ auftraten und auf die Operation zurückzuführen waren. Anschließend wurde aus diesen Komplikationen der Comprehensive-Complication-Index (CCI) für jeden einzelnen Patienten berechnet. Der CCI reflektiert dabei die Gesamtmorbidität eines einzelnen Patienten auf einer kontinuierlichen Skala von 0 (keine Komplikationen) bis 100 (Tod; [21]).

In Übereinstimmung mit der Esophagectomy Complications Consensus Group (ECCG) von 2015 wurde eine AI definiert als Vollwanddefekt im Bereich des Ösophagus, der Anastomose, der Staplernaht oder des Conduits unabhängig von der klinischen Darstellung oder der diagnostischen Methode [13]. Eine computertomographische und endoskopische Diagnostik zum Ausschluss einer AI wurde in beiden Gruppen abhängig von klinischen oder laborchemischen Indikatoren im postoperativen Verlauf eingeleitet; eine routinemäßige Ösophagogastroskopie ohne Verdachtskriterien erfolgte nicht.

Ein „textbook outcome“ wurde definiert als R0-Resektion ohne Konversion, eine Entfernung von ≥15 Lymphknoten, keine Komplikationen mit einem CDC ≥IIIa, keine Reinterventionen oder -operationen, keine Wiederaufnahme auf eine Intensivstation, eine Krankenhausverweildauer von ≤21 Tagen, keine Krankenhauswiederaufnahme oder Mortalität innerhalb von 90 Tagen postoperativ [12].

### Propensity-Scoring und statistische Auswertung

Zur Etablierung einer Vergleichsgruppe zur oben beschriebenen RAMIE-Gruppe wurde ein 1:1-PSM mit der historischen Kontrollgruppe durchgeführt, welche vollständig oder zumindest thorakal offen operiert wurde. Folgende Variablen wurden hierbei zur Berechnung des Propensity-Scores mittels Regressionsmodell genutzt: Alter, Geschlecht, Body-Mass-Index (BMI), Risikofaktoren (Rauchen, Alkohol, Diabetes), neoadjuvante Therapie, Histologie sowie pT- und pN-Stadium. Anschließend wurden gemachte Paare mithilfe der „Nearest-neighbor“-Methode und einer Breite von 0,1 gebildet.

Für die statistische Auswertung der Unterschiede zwischen den beiden Gruppen wurde der exakte Test nach Fisher genutzt. Bei metrischen, normalverteilten Variablen erfolgte die Analyse mit dem Student’s T‑Test, bei nichtparametrisch verteilten Variablen mithilfe des Mann-Whitney-U-Tests. Es erfolgte eine uni- und multivariate Analyse mittels binärer logistischer Regression bez. der Endpunkte AI und 90-Tage-Mortalität. Bei einem *p* < 0,1 in der univariaten Analyse wurden die Variablen der multivariaten Analyse zugeführt. Das Signifikanzniveau wurde bei *p* = 0,05 festgelegt. Die gesamte statistische Auswertung erfolgte in SPSS 25 (SPSS Statistics, v25.0.0.2, IBM Corporation, Armonk, New York).

## Ergebnisse

### Patientencharakteristika und histopathologische Ergebnisse

Seit Umstellung des perioperativen Managements im Juli 2018 wurden bis September 2020 insgesamt 80 Patienten mittels (h)RAMIE in unserer Klinik operiert. Nach PSM konnten 75 Patienten je Gruppe in die Analyse eingeschlossen werden. Die beiden Gruppen unterschieden sich in den entsprechenden Variablen Alter, Geschlecht, BMI, Rauchen, Alkohol, Diabetes mellitus, ASA(American Society of Anesthesiologists)-Score, neoadjuvante Therapie, Histologie, pT- und pN-Stadium nicht signifikant voneinander (Tab. 1). Das mediane Alter betrug 62 (RAMIE) bzw. 63 (Kontrolle) Jahre und der BMI lag im Median bei 25,9 kg/m^2^. Die häufigste Operationsindikation war ein Adenokarzinom (67 % [RAMIE] bzw. 60 % [Kontrolle]) gefolgt von einem Plattenepithelkarzinom des Ösophagus (39 % bzw. 33 %) meistens in einem T3-Stadium (31 % bzw. 35 %). Etwa 24 % der Patienten erhielten eine primäre Resektion ohne neoadjuvante Therapie. Die mediane Anzahl der entnommenen Lymphknoten betrug 22 (RAMIE) bzw. 21 (Kontrolle). In der Kontrollgruppe zeigten insgesamt 4 Patienten eine mikroskopische Tumorinfiltration (R1-Status), hingegen nur 1 Patient in der RAMIE-Gruppe (*p* = 0,183).

**Table 1:** 

	Offen	RAMIE	*p*-Wert
	*n* = 75	*n* = 75	
*Alter (Jahre) (Median (IQR))*	63,2 (55,8–71,7)	62,3 (57,6–68,7)	0,658^b^
*Geschlecht (n (%))*			0,401^a^
Weiblich	10 (13,3)	8 (10,7)	
Männlich	65 (86,7)	67 (89,3)	
*BMI (kg/m*^*2*^*) (Median (IQR))*	25,9 (23,2–29,0)	25,9 (23,2–30,0)	0,635^c^
*Rauchen (n (%))*	31 (41,3)	33 (44,0)	0,434^a^
*Alkohol (n (%))*	20 (26,7)	18 (24,0)	0,426^a^
*Diabetes mellitus (n (%))*	22 (29,3)	21 (28,0)	0,500^a^
*ASA (n (%))*			0,803^a^
1	0 (0,0)	0 (0,0)	
2	29 (38,7)	25 (33,3)	
3	45 (60,0)	49 (65,3)	
4	1 (1,3)	1 (1,3)	
*Neoadjuvante Vorbehandlung (n (%))*			0,179^a^
Keine	18 (24,0)	17 (22,7)	
Chemotherapie	17 (22,7)	27 (36,0)	
Radiochemotherapie	40 (53,3)	31 (41,3)	
*Histologie (n (%))*			0,498^a^
Adenokarzinom	45 (60,0)	50 (66,7)	
Plattenepithelkarzinom	29 (38,7)	25 (33,3)	
Sonstiges	1 (1,3)	0 (0,0)	
*pT-Stadium (n (%))*			0,824^a^
0	10 (13,3)	13 (17,3)	
1	20 (26,7)	24 (32,0)	
2	17 (22,7)	14 (18,7)	
3	26 (34,7)	23 (30,7)	
4	2 (2,7)	1 (1,3)	
*pN-Stadium* *(n (%))*			0,270^a^
0	45 (60,0)	44 (58,7)	
1	12 (16,0)	19 (25,3)	
2	10 (13,3)	9 (12,0)	
3	8 (10,7)	3 (4,0)	
*pM-Stadium (n (%))*			0,500^a^
0	69 (92,0)	70 (93,3)	
1	6 (8,0)	5 (6,7)	

**Fett** markiert sind signifikante Ergebnisse

*ASA* American Society of Anesthesiologists Classification, *BMI* Body-Mass-Index, *IQR* „interquartile range“, *RAMIE *roboter-assistierte minimal-invasive Ösophagektomie

^a^Fisher’s Exact Test, ^b^t‑Test, ^c^Mann-Whitney‑U

### Operationskennzahlen

In der RAMIE-Gruppe wurden 62 Patienten (82,7 %) sowohl am Abdomen als auch am Thorax unter Roboterassistenz operiert (totale RAMIE). Bei 11 Patienten (14,7 %) erfolgte der abdominelle Teil offen und bei 2 Patienten (2,7 %) laparoskopisch (hRAMIE; Tab. 2). Bei 3 Patienten (4,0 %) wurde auf eine offene Operation konvertiert. Die Gründe hierfür waren Blutung, Adhäsionen nach Bestrahlung und fehlende Übersicht bei nicht kollabierender rechter Lunge. In der Vergleichsgruppe wurden 86,7 % aller Operationen vollständig offen durchgeführt, nur bei 13,3 % der Patienten wurde ein laparoskopischer Ansatz im abdominellen Teil gewählt.

**Table 2:** 

	Offen	RAMIE	*p*-Wert
	*n* = 75	*n* = 75	
*Operation Thorax (n (%))*			**<0,001**^**a**^
Offen	75 (100,0)	0 (0,0)	
Roboterassistenz	0 (0,0)	75 (100,0)	
*Operation Abdomen (n (%))*			**<0,001**^**a**^
Offen	65 (86,7)	11 (14,7)	
Laparoskopisch	10 (13,3)	2 (2,7)	
Roboterassistenz	0 (0,0)	62 (82,7)	
*R‑Status (n (%))*			0,183^**a**^
0	71 (94,7)	74 (98,7)	
1	4 (5,3)	1 (1,3)	
*Lymphknoten entnommen (n) (Median (IQR))*	21 (16–25)	22 (15–28)	0,856^**a**^
*Blutverlust (ml) (Median (IQR))*	500 (400–800)	300 (100–500)	**<0,001**^**b**^
*Operationszeit (min) (Median (IQR))*	339 (300–408)	490 (406–558)	**<0,001**^**b**^
*Zirkularstaplergröße (n (%))*			**<0,001**^**a**^
Keine Angabe	4 (5,3)	1 (1,3)	
≤25 mm	68 (90,6)	1 (1,3)	
≥28 mm	3 (4,0)	73 (97,3)	

**Fett** markiert sind signifikante Ergebnisse

*IQR* „interquartile range“, *RAMIE* roboter-assistierte minimal-invasive Ösophagektomie

^**a**^Fisher’s Exact Test, ^b^Mann-Whitney‑U

Die Operationszeit war mit 490 min im Median (IQR [„interquartile range“] 406–558 min) in der RAMIE-Gruppe signifikant länger als in der Kontrollgruppe mit 339 min (IQR 300–408 min, *p* < 0,001, Tab. 2). Im Gegensatz dazu war der Blutverlust in der RAMIE-Gruppe signifikant niedriger (300 vs. 500 ml, *p* < 0,001). In ca. 90 % der Fälle wurde in der Kontrollkohorte ein Zirkularstapler mit einem Diameter von ≤25 mm verwendet, hingegen in 97 % eine Staplergröße von ≥28 mm in der RAMIE-Gruppe (*p* < 0,001).

### Morbidität und Mortalität

Einen optimalen postoperativen Verlauf („textbook outcome“) zeigten 39,7 % der RAMIE-Patienten und 30,7 % der Patienten in der Vergleichskohorte (*p* = 0,196). Die Häufigkeit von Patienten mit mindestens einer schwerwiegenden Komplikation (CDC ≥IIIa) unterschied sich mit 44,0 und 46,7 % nicht signifikant in beiden Gruppen (*p* = 0,435), genauso wie die durchschnittliche Gesamtmorbidität gemessen mittels CCI (22,2 vs. 25,4, *p* = 0,233).

Werden jedoch die chirurgischen Komplikationen isoliert betrachtet, zeigte sich eine signifikant geringere Rate an postoperativer AI (8,0 %) nach RAMIE im Vergleich zur Kontrollgruppe (25,3 %, *p* = 0,004). Analog war die postoperative Wundinfektionsrate nach RAMIE signifikant niedriger (4,0 % vs. 17,3 %, *p* = 0,008). Weitere chirurgische Komplikationen wie Blutungen, postoperative Verhalte oder Passagestörungen unterschieden sich nicht signifikant (Tab. 3).

**Table 3:** 

	Offen	RAMIE	*p*-Wert
	*n* = 75	*n* = 75	
*„Textbook outcome“ (n (%))*	23 (30,7)	29 (38,7)	0,196^a^
*CDC ≥IIIa (n (%))*	35 (46,7)	33 (44,0)	0,435^a^
*CCI (Mittelwert* *±* *SD)*	25,4 (23,2)	22,2 (19,7)	0,233^b^
*Chirurgische Komplikationen (n (%))*			
Anastomoseninsuffizienz	19 (25,3)	6 (8,0)	**0,004**^**a**^
Abdominale/thorakale Verhalte	7 (9,3)	3 (4,0)	0,163^a^
Wundinfektion	13 (17,3)	3 (4,0)	**0,008**^**a**^
Blutung	3 (4,0)	1 (1,3)	0,310^a^
Passagestörung	12 (16,0)	19 (25,3)	0,113^a^
Lymphfistel	2 (2,7)	5 (6,7)	0,221^a^
*Medizinische Komplikationen (n (%))*			
Pneumonie	11 (14,7)	5 (6,7)	0,092^a^
Respiratorische Insuffizienz	27 (36,0)	18 (24,0)	0,077^a^
Pulmonale Komplikationen	33 (44,0)	22 (29,3)	**0,045**^**a**^
Bakteriämie/Sepsis	3 (4,0)	1 (1,3)	0,310^a^
Thrombembolisches Ereignis	8 (10,7)	9 (12,0)	0,500^a^
*Therapie Anastomoseninsuffizienz (n (%))*			0,637^a^
Endoskopisch/interventionell	12 (63,2)	4 (66,7)	
Operativ	7 (36,8)	2 (33,3)	
*Reoperation (n (%))*			**0,002**^**a**^
Offen	13 (17,3)	3 (4,0)	
Minimal-invasiv	0 (0,0)	5 (6,7)	
*IST-Aufenthalt*			
Postoperativ (Tage) (Median (IQR))	4,8 (3,2–6,7)	3,0 (1,0–4,6)	**<0,001**^**b**^
Insgesamt (Tage) (Median (IQR))	5,7 (3,5–10,2)	3,5 (1,1–5,8)	**<0,001**^**b**^
Wiederaufnahme (*n* (%))	20 (26,7)	12 (16,0)	0,081^a^
*Krankenhausaufenthalt (Tage) (Median (IQR))*	20 (14–37)	14 (12–22)	**<0,001**^**b**^
*90-Tage-Wiederaufnahme*	11 (14,7)	3 (4,0)	**0,023**^**a**^
*30-Tage-Mortalität*	6 (8,0)	2 (2,7)	0,138^a^
*90-Tage-Mortalität*	10 (13,3)	3 (4,0)	**0,039**^**a**^

**Fett** markiert sind signifikante Ergebnisse

*CCI* Comprehensive Complication Index, *CDC* Clavien-Dindo-Klassifikation, *IQR* „interquartile range“, *ITS* Intensivstation, *RAMIE* roboter-assistierte minimal-invasive Ösophagektomie, *SD* Standardabweichung

^a^Fisher’s Exact Test, ^b^Mann-Whitney‑U

Patienten nach RAMIE erlitten jedoch postoperativ deutlich seltener pulmonale Komplikationen wie Pneumonien oder respiratorische Insuffizienzen (29,3 % vs. 44,0 %, *p* = 0,045).

Auch die Rate an offenen Reoperationen war in der RAMIE-Gruppe signifikant geringer (4,0 % vs. 17,3 %, *p* = 0,002). Bei 5 Patienten (6,7 %) konnte eine Revision minimal-invasiv erfolgen, dabei wurde bei 3 Patienten aufgrund eines Chylothorax ein retroperitoneoskopisches Clippen des Ductus thoracicus durchgeführt; bei 1 Patient erfolgte der Verschluss einer Leckage mittels videoassistierter thorakoskopischer Chirurgie (VATS) und bei 1 Patient wurde laparoskopisch eine enterokutane Fistel saniert. In beiden Gruppen konnten eine AI in zwei Drittel der Fälle mittels endoskopischer bzw. interventioneller Verfahren wie beispielsweise einer endoluminalen Vakuumtherapie oder einer CT-gestützte Drainagenanlage austherapiert werden (Tab. 3).

Sowohl der postoperative als auch der gesamte Intensivaufenthalt war in der RAMIE-Gruppe im Median 2 Tage kürzer (*p* < 0,001); nur die Wiederaufnahmerate auf eine Intensivstation unterschied sich nicht signifikant (16,0 % vs. 26,7 %, *p* = 0,081). Die mediane Krankenhausverweildauer betrug nach RAMIE nur 14 Tage (IQR 12–22), verglichen mit 20 Tagen (IQR 14–37, *p* < 0,001) in der Kontrollgruppe.

Die 30-Tage-Mortalität war in der offenen Gruppe mit 8,0 % im Vergleich zur RAMIE-Gruppe mit 2,7 % höher (*p* = 0,138). Die 90-Tage-Wiederaufnahmerate (*p* = 0,023) und die 90-Tage-Mortalität (*p* = 0,039) waren in der RAMIE-Gruppe mit jeweils 4,0 % signifikant niedriger als in der Vergleichsgruppe mit 14,7 % bzw. 13,3 %.

Entsprechend der uni- und multivariaten Analyse der Gesamtkohorte (*n* = 157) erwies sich nur die Zuordnung in die RAMIE-Gruppe als einziger multivariat unabhängiger Faktor zur Reduzierung des Risikos, eine AI im postoperativen Verlauf zu entwickeln (Odds Ratio 0,208, 95 %-Konfidenzintervall [0,047–0,919], *p* = 0,038).

## Diskussion

Die abdominothorakale Ösophagektomie ist als komplexer Zweihöhleneingriff nach wie vor komplikationsreich und daher haben Strategien zur Reduktion der Morbidität einen hohen Stellenwert. Dabei gilt es gleichfalls, die onkologische Radikalität nicht einzuschränken. Hierbei scheint vor allem ein minimal-invasiver Ansatz im thorakalen Teil pulmonalen Komplikationen vorbeugen zu können [27]. Die Rate der pulmonalen Komplikationen schwankt zwischen den erfahrenen Zentren nach RAMIE erheblich zwischen 17 und ca. 40 % [17, 24, 25], was sicher auch an einer fehlenden einheitlichen Definition liegt. Einheitlich scheint aber eine signifikante Reduktion durch das minimal-invasive Vorgehen zu sein, wie auch in der vorliegenden Studie gezeigt werden konnte.

Zudem konnte durch den hier beschriebenen Strategiewechsel die Rate an AI signifikant auf 8 % reduziert werden. Eine vergleichbar niedrige Rate wurde unlängst von einem weiteren RAMIE-Zentrum publiziert [24]. Für die Reduktion der AI ist wahrscheinlich aber nicht allein die Roboterassistenz verantwortlich, da die erste randomisierte Studie aus den Niederlanden (ROBOT-Trial) in der RAMIE-Gruppe zwar eine Senkung der chirurgischen und pulmonalen Komplikationsrate sowie der postoperativen Schmerzen, jedoch eine AI-Rate von 24 % (13 von 54 Patienten, 20 % in der offenen Kontrollgruppe) nach zervikaler Anastomose (handgenähte End-zu-Seit-Ösophagogastrostomie) berichtete [25]. Somit ist die Roboterassistenz alleine kein Garant für eine niedrige AI-Rate. Es scheint die Frage berechtigt, ob RAMIE mit intrathorakaler Anastomose (Ivor Lewis) daher die bessere Kombination darstellt, um eine niedrige AI-Rate (auch multizentrisch) erzielen zu können. Die bisher größte Single-Center-Studie aus den USA wies eine AI-Rate von knapp 16 % nach 350 RAMIEs aus [17]. Auch wenn eine AI-Rate von 24 % überdurchschnittlich hoch erscheint, spiegelt dies durchaus realistische Zahlen nach Ösophagektomie wider [1].

Janssen et al. konnten zeigen, dass durch zusätzliche perioperative SDD-Gabe die AI-Rate nach Ivor-Lewis-MIE halbiert werden konnte [9]. Außerdem scheint eine an die anatomischen Gegebenheiten angepasste Wahl des Zirkularstaplers mit größtmöglichem Durchmesser ebenfalls die AI-Rate weiter reduzieren zu können. So war in einer Analyse von 632 Patienten nach Ivor-Lewis-Ösophagektomie die AI-Rate in der Gruppe mit einem Diameter von 28 mm um ein Drittel niedriger verglichen mit 25 mm Durchmesser, jedoch nur mit einem statistischen Trend (*p* = 0,092; [15]).

In der vorliegenden Arbeit berichten wir von dem Ansatz, diese möglicherweise sinnvollen Einzelmaßnahmen zur Senkung der Morbidität in einem Strategiewechsel zu kombinieren. Wir haben dadurch mittlerweile ein hochstandardisiertes Management von Ösophagektomien erzielt, mit sehr konstanten Ergebnissen über die Zeit. Die berichteten Eingriffe wurden durch 3 Operateure (aktuell 4) vorgenommen. Es zeigten sich signifikante Vorteile hinsichtlich geringerer Anastomoseninsuffizienz, geringerer pulmonaler Komplikationen, Wundinfekten, Reoperationen, Mortalität und eines kürzeren Intensiv- und Krankenhausaufenthalts.

### Limitationen

Es handelt sich um eine retrospektive Vergleichsstudie und daher sind die Ergebnisse entsprechend zu bewerten. Die RAMIE-Gruppe inkludiert 11 Patienten mit einer Hybridoperation (abdominal offen, thorakal robotisch), was als zusätzliches Bias berücksichtigt werden muss. Darüber hinaus wurden die Daten zur Lernkurvenentwicklung nicht präsentiert, da eine separate Auswertung angestrebt wird. Jedoch war die Reduktion der o. g. Outcomeparameter konstant über den Beobachtungszeitraum. Die vorgestellten Daten geben keinen Aufschluss darüber, welche Einzelkomponenten (Roboterassistenz, minimal-invasiver Eingriff, Staplergröße, ICG, SDD) einen entscheidenden Einfluss auf die o. g. Outcomeparameter haben.

## Fazit für die Praxis

Insgesamt lässt sich festhalten, dass durch den Strategiewechsel von offener Ösophagektomie zu einem standardisierten roboter-assistierten minimal-invasiven Ösophagektomie(RAMIE)-basierten, perioperativen Management

- eine exzellent niedrige Anastomoseninsuffizienzrate von ca. 8 % erreicht werden kann,
- Wundinfektionen und pulmonale Komplikationen seltener auftreten,
- seltener offene Reoperationen notwendig sind,
- Intensiv- und Krankenhausaufenthalt signifikant verkürzt sind und
- die 90-Tage-Mortalität reduziert ist.

Die Studie ist damit ein Beleg dafür, dass die klinisch signifikanten Vorteile der RAMIE-Technik sich in weiteren Zentren auf hohem Niveau in einem standardisierten Setting reproduzieren lassen und Patienten zugutekommen können.
